# Supplementary material for: Diffusion Kurtosis Imaging—A Superior Approach to Assess Tumor–Stroma Ratio in Pancreatic Ductal Adenocarcinoma
Source: Cancers (Basel). 2020 Jun 22;12(6):1656. doi: 10.3390/cancers12061656 (PMC7352692; doi:10.3390/cancers12061656)
Supplement: Supplementary file 1 [file cancers-12-01656-s001.zip › Cancers-Supplementary-Material-2020-06-22.docx]

Article

Diffusion kurtosis imaging – a superior approach to assess tumor-stroma-ratio in pancreatic ductal adenocarcinoma.

Philipp Mayer, Yixin Jiang, Tristan A. Kuder, Frank Bergmann, Ekaterina Khristenko, Verena Steinle, Jörg Kaiser, Thilo Hackert, Hans-Ulrich Kauczor, Miriam Klauß, and Matthias M. Gaida


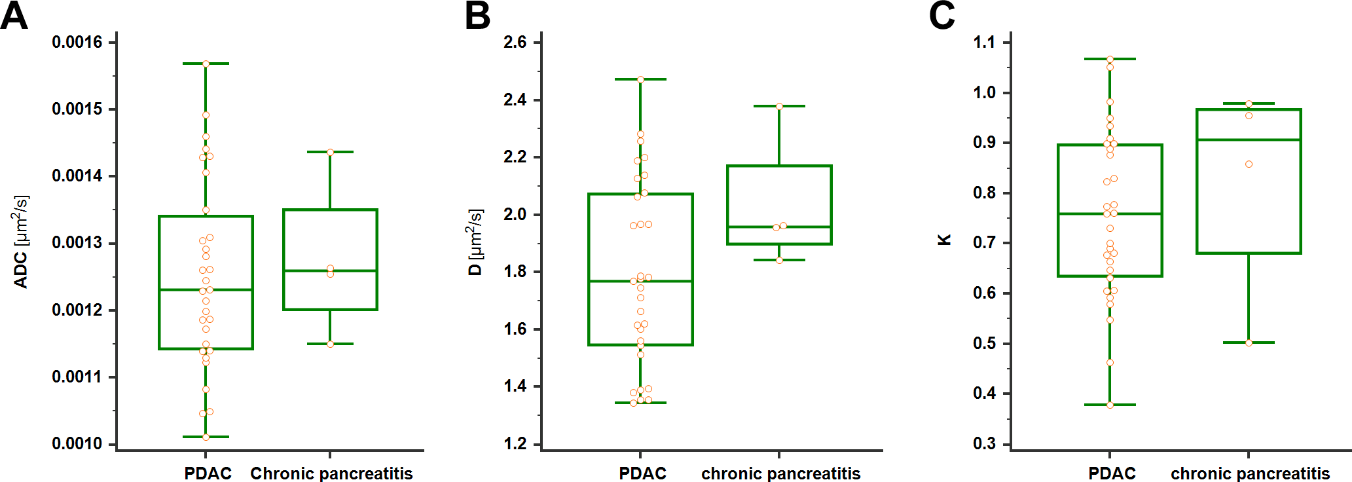


**Supplementary Figure 1.** Box-and-whisker plots of ADC, D, and K values in PDAC and chronic pancreatitis. ADC values (A), D values (B), and K values (C) were non-significantly higher in chronic pancreatitis lesions than in PDAC lesions (p ≥ 0.1949).


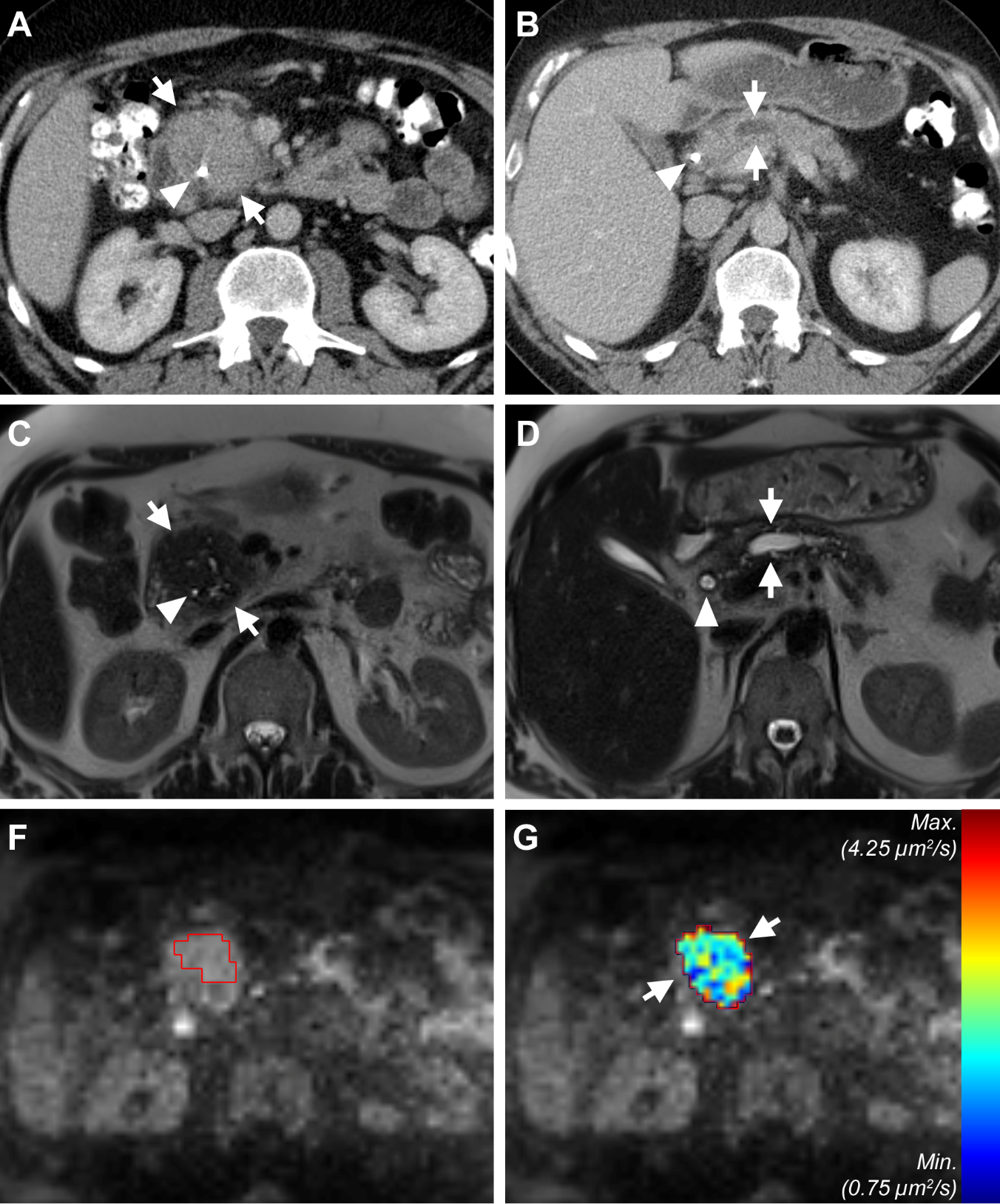


**Supplementary Figure 2.** Example pictures of a 46-year-old male patient with chronic pancreatitis. A) Axial venous phase CT image showing enlargement of the pancreatic head without calcifications (*arrows*). Stent in the distal common bile duct (*arrowhead*). B) Axial venous CT image at the level of the pancreatic body showing marked upstream dilatation of the main pancreatic duct (*arrows*). Stent in the distal common bile duct (*arrowhead*). C) Axial Half-Fourier-Acquired Single-shot Turbo spin Echo (HASTE) T2-weighted image confirming isointense enlargement of the pancreatic head (*arrows*). D) Axial T2-weighted image at the level of the pancreatic body confirming marked upstream dilatation of the main pancreatic duct (*arrows*). The common bile duct is only mildly dilated (*arrowheads*) C) Diffusion weighted image (DWI) with b-value 1000 s/mm^2^ with the volume of interest (*red*) from reader 1, avoiding major ductal structures. D) Corresponding color-coded D map. Note that the pancreatic head is colored mainly in blue and green, representing moderate D values. Mean D for both readers was 1.962 µm^2^/s.

**Supplementary Table 1.** Comparison of conventional MRI features and diffusion kurtosis imaging analysis in PDAC patients.

|  | **Tumor compared to upstream parenchyma**  (X/Y: X out of Y cases) | **Tumor compared to downstream parenchyma**  (X/Y: X out of Y cases) |
| --- | --- | --- |
| **Native T1-weighted imaging** | hypointense: 15/29 (51.7%)  isointense: 14/29 (48.3%)  hyperintense: 0/29 (0.0%) | hypointense: 8/8 (100.0%)  isointense: 0/8 (0.0%)  hyperintense: 0/8 (0.0%) |
| **Native T2-weighted imaging** | hypointense: 1/29 (3.4%)  isointense: 19/29 (65.5%)  hyperintense: 9/29 (31.0%) | hypointense: 0/8 (0.0%)  isointense: 4/8 (50.0%)  hyperintense: 4/8 (50.0%) |
| **DWI** **(b = 1000 s/mm^2^)** | hypointense: 0/29 (0.0%)  isointense: 8/29 (27.6%)  hyperintense: 21/29 (72.4%) | hypointense: 0/8 (0.0%)  isointense: 2/8 (25.0%)  hyperintense: 6/8 (75.0%) |
| **D_up-/downstream_** *minus* **D_tumor_** | ≥ 0.3 µm^2^/s: 23/29 (79.3%)  < 0.3 µm^2^/s: 6/29 (20.7%) | ≥ 0.3 µm^2^/s: 6/8 (75.0%)  < 0.3 µm^2^/s: 2/8 (25.0%) |

| 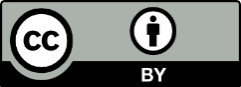 | © 2020 by the authors. Submitted for possible open access publication under the terms and conditions of the Creative Commons Attribution (CC BY) license (http://creativecommons.org/licenses/by/4.0/). |
| --- | --- |
