# Supplementary figures and images for: Diffusion Kurtosis Imaging—A Superior Approach to Assess Tumor–Stroma Ratio in Pancreatic Ductal Adenocarcinoma
Source: Cancers (Basel). 2020 Jun 22;12(6):1656. doi: 10.3390/cancers12061656 (PMC7352692; doi:10.3390/cancers12061656)

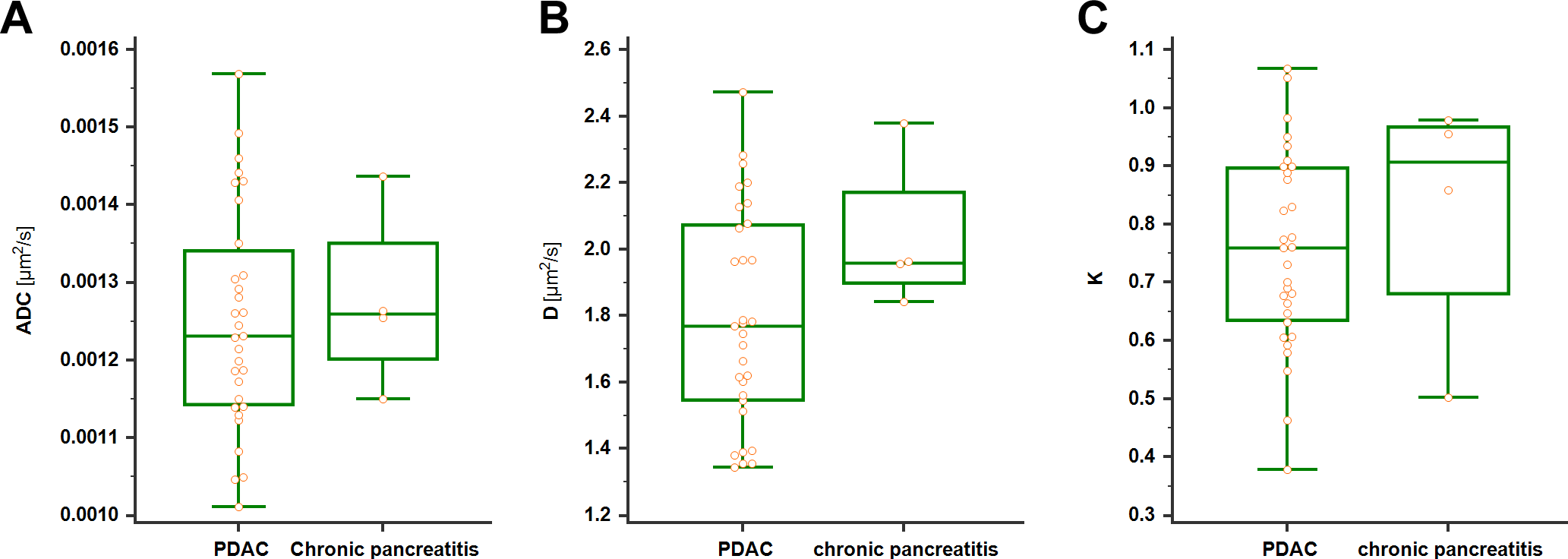

Supplement: Supplementary file 1 [file cancers-12-01656-s001.zip › Supplementary-Figure1.tif]

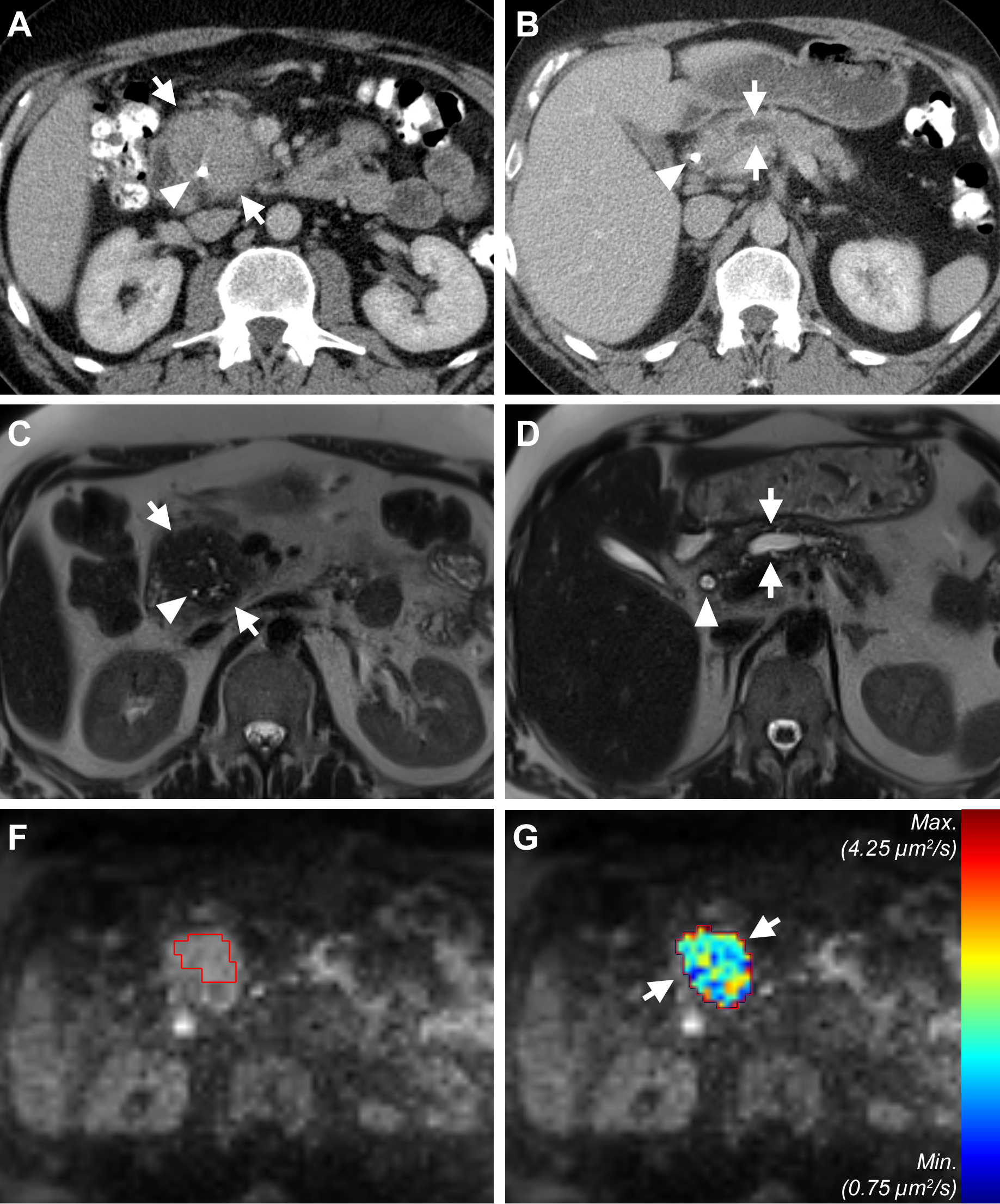

Supplement: Supplementary file 1 [file cancers-12-01656-s001.zip › Supplementary-Figure2.tif]
